# Response to: A Retrospective Forensic Review of Unexpected Infectious Deaths

**DOI:** 10.1093/ofid/ofz300

**Published:** 2019-06-27

**Authors:** Mark R Wallace, Jan C Garavaglia

**Affiliations:** 1 Infectious Disease Department, Skagit Valley Hospital, Mt Vernon, Washington; 2 Forensic Pathology, Island County, Washington

Dear Editor,

We read with interest the article by Dr. Sehgal and coauthors regarding unexpected deaths due to infection in a cohort of forensic patients from Ontario, Canada. [1]. Their article was very similar in scope and conclusions to an article that we published in 2015 [2]. Both articles retrospectively reviewed about 7000 cases of unexpected deaths and found a substantial proportion died of infections (6% in the Canadian experience and 3.4% in our study from central Florida). Gram-positive organisms (*Staphylococcus aureus, Staphylococcus pneumoniae*, and *β* hemolytic streptococci) were the most frequently identified pathogen in both forensic series, and pneumonia the most lethal infection. Pyelonephritis was an infrequent cause of death (5% vs 2%). The proportions of infectious unexplained deaths related to illicit drug use were also remarkably similar (17% vs 24%) in the 2 studies. Our study differed in some important respects, however. It was a longer look at a smaller geographic area (Orlando, Florida, and surrounding area, population ~2 million, vs 14 million for Ontario, Canada) but had more standardization, as all death investigations and autopsies were done out of 1 medical examiner’s office rather than through multiple coroners’ offices.

We commend Sehgal and colleagues for their hard work and advancement of our understanding of infectious diseases in forensics.
